# Red Grape Pomace as a Valuable Source of Biomolecules With Inhibitory Effect on Pro‐Inflammatory and Metabolic Syndrome‐Associated Enzymes: Eco‐Friendly Extraction and Mechanism of Inhibition

**DOI:** 10.1002/fsn3.71938

**Published:** 2026-05-24

**Authors:** Nicoleta Balan, Silviu Măntăilă, Ilir Mërtiri, Elisabeta Irina Geana, Iuliana Aprodu, Gabriela Râpeanu, Nicoleta Stănciuc

**Affiliations:** ^1^ Faculty of Food Science and Engineering Dunărea de Jos University of Galati Galați Romania; ^2^ National Research and Development Institute for Cryogenic and Isotopic Technologies Râmnicu Vâlcea Romania

**Keywords:** antidiabetic, anti‐inflammatory, eutectic solvents, inhibition, polyphenols, red grape pomace

## Abstract

In this study, the interaction models between an extract of red grape pomace obtained by extraction with eutectic solvents and three enzymes, responsible for pro‐inflammatory (lipoxygenase) and metabolic syndrome responses (α‐amylase and α‐glucosidase), were systematically investigated through experiments and computational simulations. The chromatographic analysis allowed the identification of thirty‐two polyphenolic compounds in the extract, predominantly phenolic acids (73%), with a total polyphenol content of 575.58 mg/kg. The main identified compounds were vanillic acid, azelaic acid, chlorogenic acid, suberic acid, abietic acid, phlorizin, polydatin, and t‐resveratrol. SwissADME and ProTox‐3.0 bioinformatic tools were employed to assess the absorption, distribution, metabolism, and excretion, highlighting that 23.23% of the identified compounds may exert good oral bioavailability in humans and neuroprotective or cognitive‐enhancing effects within the central nervous system. A high inhibition rate of 99% for amylase and glucosidase and 82% for lipoxygenase was suggested. The thermodynamic analysis suggested that hydrogen bonding, van der Waals, and hydrophobic forces primarily drove the interactions. The molecular docking results revealed details on the potential mechanisms involved in the inhibition of the metabolic syndrome‐associated enzymes exerted by the phenolic compounds from red grape extract. Different phenolic compounds were identified to attach to the active site of the enzymes, interfering with their activity. The anti‐diabetic activity might be assigned to vanillic acid and suberic acid, which bind to α‐amylase and α‐glucosidase respectively, whereas suberic acid and resveratrol appeared to be responsible for the direct inhibitory mechanism of lipoxygenase.

## Graphical abstract

Superposition of the top scoring enzyme‐ligand models resulting from the *in silico* docking tests: complexes formed by α‐amylase (a), α‐glucosidase (b), and lipoxygenase (c) with vanillic acid (magenta), azelaic acid (red), phlorizin (yellow), cis‐polydatin (green), chlorogenic acid (orange), suberic acid (black), abietic acid (blue), t‐resveratrol (pink). The enzymes, ligands, and catalytic amino acids are represented in NewCartoon, licorice, and Van der Waals style, respectively, by using the VMD software (Humphrey et al. 1996).

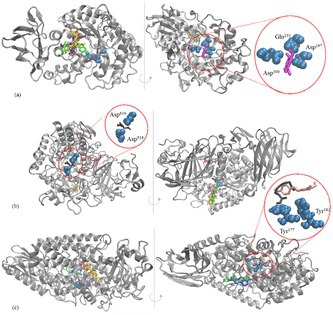

## 1 Introduction

It is well known that in the food chain, most technological processes are represented by successive linear sequences of operations, known as “take, make, dispose” (Ponticelli et al. 2025), thus generating enormous quantities of waste and by‐products, which most often end up dispersed in landfills or incinerators (Lavelli 2021). In general, the actual trend is oriented towards the *zero waste* concept, which underlies the efforts to design more sustainable food systems by improving the waste and by‐products way of management, as they are seen as valuable and generous resources of compounds with positive health effects. One of the agrifood sectors that urgently requires the development of efficient waste management technologies is the wine chain, which has significant economic implications at a global level and represents an important source of waste and valuable by‐products (Crescente et al. 2023).

Out of the global production of 80.1 million tonnes of grapes in 2022, about 5.6 million tonnes were not valorised, whereas 37.3 million tonnes were processed through pressing (International Organisation of Vine and Wine 2022). In wine production, grape processing results in various wastes, mainly pomace, stems, and residual lees. Approximately 10%–30% of the weight of processed grapes is given by a solid organic waste, known as grape pomace (GP), which mainly contains skins and seeds. In the current practices, GP is used as animal feed; however, GP may be used as raw material for various alternatives, in particular for the extraction of molecules with significant health impact, mainly polyphenols, such as tannins, phenolic acids, flavan‐3‐ols, stilbenes, flavonols, anthocyanins, and chalcones. Various studies reported specific health‐related benefits of these biomolecules, such as antioxidant, anti‐inflammatory, anticancer, antimicrobial, cardioprotective, and anti‐aging factors (Abouelenein et al. 2023; Cisneros‐Yupanqui et al. 2023; Coluccia et al. 2020).

Recently, the concept of green extraction techniques was developed, being applicable for recovering the active compounds from byproducts, as they meet the Environmental Protection Agency (EPA) standards set, referring especially to the use of safe solvents, whereas designing solutions for energy efficacy (https://www.epa.gov/greenchemistry/pubs/about_gc.html).

The environmental impact when extracting secondary metabolites from food‐chain by‐products is of great scientific interest, taking into account current pollution reduction guidelines. Misra et al. (2023) suggested that improper disposal of waste and by‐products can release phenolic compounds into the soil, which leads to significant ecological repercussions on plant development, especially during the germination stage. In addition to secondary metabolite extraction techniques, the choice of solvent is of particular importance in order to minimize the environmental emissions and to preserve human health, when using the corresponding extracts for nutraceutical, food, or cosmetic applications (Ponticelli et al. 2025). Given the analytical processes involved in extracting high‐value, bioactive compounds from natural matrices with targeted function, the use of biodegradable, renewable, low‐toxicity, and more efficient solvents aligns with the 12 principles of Green Chemistry. In this context, the design of natural deep eutectic solvents (NaDES) as potential alternatives to conventional solvents should be taken into consideration. In principle, NaDES consist of a mixture of hydrogen donors and hydrogen acceptors, which is differentiated by the lower melting point compared to that of their individual components, due to the generation of intermolecular hydrogen bonds (Meng et al. 2018).

It has been demonstrated that changes in food habits and sedentary lifestyle are associated with obesity, insulin resistance, hyperlipidemia, and hypertension, aspects that have led to an increase in the prevalence of metabolic syndrome and proinflammatory diseases worldwide (Papaioannou et al. 2022). For example, type‐2 diabetes is a chronic condition characterized by insulin resistance and ß‐cell failure, resulting from poor lifestyle habits that interact with an underlying genetic susceptibility. It has been suggested that inhibition of α‐amylase and α‐glucosidases is an effective alternative in both preventing and treating type‐2 diabetes through reducing postprandial hyperglycaemia. Polyphenols, especially anthocyanins, are biomolecules well known for their anti‐inflammatory activity, which explains the various biological effects related to the antidiabetic and anticancer activities, and their role in cardio‐brain prevention (Liu et al. 2023).

While the literature associates dietary specialized biomolecules from GP with a reduced risk of type 2 diabetes and proinflammatory processes, the mechanisms of inhibition are not sufficiently detailed. Therefore, new knowledge is needed to deepen the kinetics and thermodynamics of the interactions between small molecules and enzymes with metabolic implications, as new natural inhibitors with applications in clinical medicine, chemistry, food science and biotechnology, which would counteract the side effects of drugs applied in current practice, especially gastrointestinal dysfunctions due to non‐specific inhibitory activity (Kadouh et al. 2016).

Therefore, the main objective of this study was to investigate the mechanism of metabolic syndrome associated and proinflammatory enzymes inhibition induced by the biomolecules extracted from red grape pomace (RGP) using NaDES. With this purpose, the extract was obtained by using NaDES represented by a combination of choline chloride, lactic acid, and ultrapure water (ratio of 1:2:1), after the optimization and validation ultrasound‐assisted extraction step (Balan et al. 2025). The theoretical and computational predictions for the absorption, distribution, metabolism, and excretion (ADME) as well as the oral and nutritional toxicity of the phytochemical compounds identified in the NaDES extract were performed using the SwissADME online and the ProTox‐3.0 online server. Further, the interactions of the extract with α‐amylase, α‐glucosidase, and lipoxygenase were investigated in order to obtain an in‐depth molecular understanding of the binding mechanism by using spectrofluorimetric and molecular docking methods. The number of binding sites, binding parameters, and thermodynamic parameters for the complexation process were obtained from the analysis of spectrofluorimetric titration data. The binding site particularities and the potential interference of the ligands' attachment with the specific activity of the investigated enzymes were assessed upon running molecular docking simulations. The results were correlated with the half‐maximal inhibitory concentration (IC50) and compared with the corresponding values on the most common drugs used in current practices. The results obtained in this study are promising for the future development of food‐derived inhibitors from grape pomace for preventing type‐2 diabetes and proinflammatory diseases.

## 2 Materials and Methods

### 2.1 Chemical and Reagents

Folin–Ciocâlteu reagent, choline chloride ≥ 98% ((2‐hydroxyethyl)trimethylammonium chloride), lactic acid 90%, sodium phosphate dibasic heptahydrate ≥ 98%, sodium phosphate monobasic monohydrate ≥ 98%, sodium chloride ≥ 99%, 4‐Nitrophenyl α‐D‐glucopyranoside ≥ 99%, linoleic acid ≥ 99%, quercetin ≥ 98% (HPLC), α‐amylase from porcine pancreas (~50 U/mg), α‐glucosidase from *Saccharomyces cerevisiae* (≥ 10 units/mg protein), lipoxygenase from *Glycine max* (soybean) (≥ 50,000 units/mg solid) were purchased from Sigma‐Aldrich (Millipore Sigma, Steinheim, Germany). Acarbose, 95% was purchased from Acros Organics (China), 3,5‐Dinitrosalicylic acid (DNS), starch soluble (from potatoes) were purchased from Lach‐Ner. S.r.o (Czech Republic), methanol for HPLC, ≥ 99.9%, was purchased from Honeywell (Seelze, Germany) and sodium carbonate was purchased from S.C. Chemical Company SA (Iasi, Romania).

### 2.2 Plant Material

The unfermented red grape pomace (RGP) ( *Vitis vinifera* L.) from the Fetească Neagră and Merlot varieties was kindly provided by Bratu winery, located in Odaia Manolache village, Vânători commune, Galați County, Romania (45°33′27.5182″ N, 28°0′21.7552″ E). The raw material was stored at –20°C in refrigerated containers until use. The RGP were preserved by drying in a hot air convection (Stericell 111 ECO Hot AIR Sterilizer; MMM Medcenter Einrichitungen GmbH, München, Germany) at a moderate temperature (35°C) up to a moisture content of 8%. Subsequently, the dried material was ground using an electric grinder (Coffee grinder Heinner HCG‐150SS; Heinner, Network One Distribution, Bucharest, Romania) and stored in vacuum‐sealed bags under refrigeration conditions (4°C).

### 2.3 Ultrasound‐Assisted Extraction (UAE)

The UAE using NaDES as solvents was performed at optimum parameters, as explained in our previous study (Balan et al. 2025). In this study, the solvent was represented by a combination of choline chloride, lactic acid, and water in a molar ratio of 1:2:1, whereas the UAE was performed at a temperature ranging from 40°C to 60°C; extraction time between 30 and 60 min; and the solid–liquid (S/L) ratio of NaDES (1:10–1:20 mL). From the data reported earlier (Balan et al. 2025), the optimum parameters for RGP bioactive extraction were: temperature of 60°C, for 60 min of extraction and S/L ratio of 1:10 mL. The models were validated, whereas the extraction was repeated three times in order to exhaust the RGP in bioactive.

### 2.4 Total Polyphenolic Content (TPC)

The modified Folin–Ciocalteu colorimetric technique, described by Serea et al. (2023), was used to quantify the TPC in the NaDES extract. In brief, 200 μL of diluted extract (1:5) was mixed with 15.8 mL ultrapure water and 1 mL of Folin–Ciocalteu reagent and allowed to react for 10 min. Further, 3 mL of 20% Na_2_CO_3_ was added, and the mixture was kept in a dark place for 60 min at 25 C. The absorbance at the wavelength of 765 nm was measured using a spectrophotometer (Biochrom; Libra 22 UV/Visible Spectrophotom‐eter; 177 Holliston, MA, USA). Measurements were made in triplicate for each sample, and the results obtained were expressed in milligrams of gallic acid equivalents per mL (mg GAE/mL).

### 2.5 Liquid Chromatography High Resolution Mass Spectrometry (HRMS) Analysis of Phenolic Compounds

For the determination of phenolic compounds profile by HRMS analysis, an aliquot of the sample was dissolved with 1 mL mixture of water: methanol = 80:20, filtered to 0.45 μm hydrophilic membrane filter and then subjected to the instrumental analysis. The identification and quantification of the phenolic compounds in the extract were performed by using an ultra high‐performance liquid chromatograph UltiMate 3000 (UHPLC) (ThermoFisher Scientific, Bremen, Germany), coupled to a Q Exactive Focus mass spectrometer, Focus Hybrid Quadrupole—OrbiTrap (Thermo Fisher Scientific) equipped with HESI. Chromatographic separation of phenolic compounds was performed on a Kinetex C18 column (100 × 2.1 mm; 1.7 μm particle diameter) maintained at 30°C, under a gradient elution of two mobile phases, A (0.1% formic acid in water) and B (0.1% formic acid in methanol), at flow rates of 0.3 and 0.4 mL/min, as previously optimized (Onache et al. 2022). The mass spectrometer was operated in negative mode, in a range between 75 and 1125 m/z, at a resolution of 70,000, under the previously optimized HESI‐source parameters (spray voltage 2.8 kV, capillary temperature 320°C, auxiliary gas heater temperature 413°C, sheath and auxiliary gas flow (N_2_) 48 and 11 arbitrary units) (Onache et al. 2022). Identification and quantification of the phenolic compounds from the extract were performed according to their spectral characteristics: mass spectra, accurate mass, and characteristic retention time against external standard solutions. Fragmentation studies by a data‐dependent scan with collision‐induced dissociation (CID) were performed in order to confirm the identified compounds. Compound quantification was performed based on the external standard method, with calibration standards between 25 and 2000 μg/L for each of the phenolic compounds, obtained by serial dilution with methanol of the 10 mg/L calibration standard mixture. All stock and working solutions were stored in the dark at 4°C. The results were expressed in mg/kg of extract. Xcalibur software (Version 4.1) was used for instrument control, data acquisition and data analysis.

### 2.6 Bioinformatic Predictions of ADME and Toxicity of the Extract Compounds

The theoretical and computational predictions for the absorption, distribution, metabolism, and excretion (ADME) as well as the oral and nutritional toxicity of the phytochemical compounds identified in the NaDES extract were performed using the SwissADME online server (swissadme.ch; accessed on 19 February 2026) (Daina and Zoete 2016), and the ProTox‐3.0 online server (tox.charite.de; accessed on 19 February 2026) (Banerjee et al. 2024). The encoding molecular structures (SMILES) of the phytochemical compounds were obtained from the PubChem online database (pubchem.ncbi.nlm.nih.gov; accessed on 19 February 2026).

### 2.7 α‐Amylase Inhibitory Assay

The efficiency of the optimized extracts in inhibiting α‐amylase was tested using the protocol described by Mahdi et al. (2020) and Serea et al. (2022) with some modification. A volume of 200 μL of enzyme solution (prepared by dissolving 1 g of α‐amylase from porcine pancreas in 13 mL of 20 mM PBS, pH = 6.9, containing 6 mM sodium chloride) was mixed with 200 μL of different diluted extract solution (concentration expressed in mg GAE/mL) and incubated for 10 min at 37°C. Subsequently, 200 μL of starch solution (0.5% w/v) was added, and the mixture was further incubated for 15 min at 37°C. Further, 400 μL of DNS reagent was added, and the mixture was placed in a boiling water bath for 5 min, followed by rapid cooling under a water jet. The samples were then diluted with 1 mL of distilled water, and the absorbance was measured at 540 nm using a UV–VIS spectrophotometer (Biochrom; Libra 22 UV/Visible Spectrophotometer; 177 Holliston, MA, US). Acarbose was used as control, following the same protocol. To calculate the percentage inhibition of α‐amylase activity, the Equation 1 was applied. To determine the sample concentration (extract/acarbose) responsible for inhibiting 50% of the α‐amylase activity, the IC_50_ value was calculated according to the logarithmic regression equation model based on the plotted data.

(1) $$I\;\left(\%\right)=\frac{\left(A_{\text{control}}-A_{\text{sample}}\right)}{A_{\text{control}}}$$

where *I* is the percentage of inhibition activity, *A*_control_ is the absorbance value obtained when the sample dilution is replaced with PBS (representing 100% enzyme activity), *A*_sample_ is the absorbance value obtained from the tested diluted extract solution. The logarithmic regression equation model based on the plotted data was applied to determine the IC_50_ (mg GAE/mL) of extract and acarbose (mg/mL).

### 2.8 α‐Glucosidase Inhibitory Assay

To analyze the inhibitory activity of the extract on α‐glucosidase, the protocol described by Serea et al. (2022) was used with minor modifications. A volume of 50 μL of α‐glucosidase from *Saccharomyces cerevisiae* (1 U/mg in 0.1 M PBS, pH = 6.9) was pre‐incubated with 100 μL of different diluted extract solution (concentration expressed in mg GAE/mL) at 37°C for 10 min. By the addition of 100 μL substrate, 25 mM *p*‐nithophenyl‐α‐D‐glucopyranizide and 1600 μL 0.1 M PBS, pH = 6.9, the enzyme reaction was initiated. After incubation at 37°C for 30 min, 800 μL of 0.2 M Na_2_CO_3_ was added to stop the enzymatic reaction. The absorbance of the samples was measured at 405 nm using a UV–VIS spectrophotometer (Biochrom; Libra 22 UV/Visible Spectrophotometer; 177 Holliston, MA, US). Acarbose was used as control. Equation 1 was used to calculate the percentage inhibition, and the logarithmic regression equation model based on the plotted data was applied to determine the IC_50_ (mg GAE/mL) of extract and acarbose (mg/mL).

### 2.9 Lipoxygenase Inhibitory Assay

To determine the inhibitory effect of the extracts on lipoxygenase, the protocol described by Serea et al. (2022) was used, with some modifications. A volume of 50 μL of enzyme solution (1 mg/mL lipoxygenase from *Glycine max* (soybean), dissolved in PBS 0.1 M, pH = 9.0) and 50 μL of different diluted extract solution (concentration expressed in mg GAE/mL) were incubated for 10 min at 37°C. Subsequently, 50 μL of substrate (0.05 mM linoleic acid, dissolved in PBS 0.1 M, pH = 9.0) was added, and the mixture was incubated for 20 min at 37°C. Finally, 2 mL of PBS 0.1 M, pH = 9.0 were added to the reaction mixture. Absorbance was measured at 234 nm using quartz cuvettes and a UV–VIS spectrophotometer (Biochrom; Libra 22 UV/Visible Spectrophotometer; 177 Holliston, MA, US). The inhibition was calculated based on Equation 1. Quercetin was used as control, and the results were expressed in IC_50_ values (mg GAE/mL).

### 2.10 Fluorescence Spectroscopy

The intrinsic fluorescence emission spectra were obtained using a LS‐55 Luminescence Spectrometer (Perkin Elmer, Waltham, MA, USA), as described by Aprodu et al. (2020). In a typical experiment, 0.1 mL of 5 mg/mL of enzyme solutions in phosphate buffer (0.1 M) at different pH values, according to the optimum activity of the enzymes (7.4 for α‐amylase, 6.9 for α‐glucosidase and 8.0 for lipoxygenase), was placed into the cuvette. A volume of 2 mL (α‐amylase and lipoxygenase) or 3 mL (α‐glucosidase) of phosphate buffer at corresponding pH was added, followed by manual titration by successive additions of extract stock solution (increasing concentration from 0 to 1.2310^−6^ Mol/L GAE). The fluorescence intensities were corrected for dilution effects before they were considered for data analysis.

The fluorescence quenching mechanism was described by the Stern‐Volmer equation, and the quenching constants of experimental data were obtained by regression analysis (Aprodu et al. 2020). The binding constants (k_b_), the number of binding sites (n), and thermodynamic parameters were estimated according to Dumitraşcu et al. (2016).

### 2.11 Heat Treatment

Plastic tubes (1 cm diameter) were filled with 0.15 mL of enzyme solutions. The samples were heated at temperatures of 37°C, 45°C, 50°C, and 55°C for 10 min using a thermostatic water bath (Digibath‐2 BAD 4, Raypa Trade, Barcelona, Spain). The heated solutions were then cooled by introducing the tubes in ice water, such as to avoid further thermal denaturation.

### 2.12 Molecular Docking Tests

The molecular docking tests were further used to check the binding particularities of the main polyphenols identified in the red grape pomace, by the pro‐inflammatory and metabolic syndrome‐associated enzymes. The molecular docking tests were carried out by means of AutoDock Vina on SwissDock (Eberhardt et al. 2021; Bugnon et al. 2024). The refined α‐amylase (6Z8L.pdb; Axer et al. 2021), α‐glucosidase (5NN5.pdb; Roig‐Zamboni et al. 2017), and lipoxygenase (3O8Y.pdb; Gilbert et al. 2011) models, selected from the RCSB Protein Data Bank, served as receptors for the following ligands: vanillic acid, azelaic acid, phlorizin, cis‐polydatin, chlorogenic acid, suberic acid, abietic acid, t‐resveratrol. The ligands used in docking tests were selected based on the chromatography results. For each type of enzyme‐ligand complex, the top scoring model, characterized by the highest affinity, as indicated by AutoDock Vina ranking, was further in‐depth characterized using the PDBePISA tools (Krissinel 2010) and VMD 1.9.3. software (Humphrey et al. 1996).

### 2.13 Statistical Analysis

Microsoft Excel for Microsoft 365, MSO version 2412, build 16.0.18324.20240 (Microsoft, Redmond, WA, USA) was used for statistical processing of the data obtained, and the results were expressed as mean ± standard deviation. The data were statistically processed using the One‐Way ANOVA method, using the Tukey test. Values that do not share the same letter indicate statistically significant differences, with *p*‐value < 0.05.

## 3 Results and Discussion

### 3.1 Polyphenolic Profile of the Extract

The extract obtained by UAE using NaDES as extraction solvent contained 3.80 ± 0.01 mg GAE/mL. A detailed polyphenolic profile was obtained by using U‐HPLC (Table 1). As it can be observed in Table 1, the chromatographic analysis allowed the identification of thirty‐two polyphenolic compounds, predominantly phenolic acids (73%), with a total polyphenol content of 575.58 mg/kg. Regarding the phenolic acids, the main compound was vanillic acid (236.36 mg/kg), followed by azelaic acid (50.09 mg/kg), chlorogenic acid (31.42 mg/kg), suberic acid (30.94 mg/kg), and abietic acid (25.01 mg/kg). As regarding the flavonoids, phlorizin was found in a higher concentration of 47.19 mg/kg, whereas the main stilbenes found were polydatin (32.05 mg/kg) and t‐resveratrol (20.66 mg/kg). From the data reported, it seems that the NaDES variant used in this study allowed us to enhance the extraction of phenolic acids when compared with flavonoids and anthocyanins. Therefore, when using HPLC technique, Balan et al. (2025) identified only nine bioactives, namely phenolic acids (gallic acid, caffeic acid, protocatechuic acid, ellargic acid, and chlorogenic acid), flavonoids (epigallocatechin, catechin, and rutin trihydrate), and terpenoids (cafestol). Epigallocatechin and catechin were the main compounds with the highest concentration in the NaDES extract, with values of 91.77 ± 0.02 and 4.51 ± 0.01 mg/mL, respectively. Moutinho et al. (2023) reported a concentration of polyphenolic compounds of 2.407 ± 0.032 mg/mL, with phenolic acids in a concentration of 1.212 ± 0.015 mg/mL. These authors identified by HPLC–DAD–ESI–MS/MS in extracts obtained at 60°C using 3.5% HCl and 70% ethanol from the variety “Touriga Nacional” and “Sousão” fourteen compounds among which were found: 3‐*O*‐caffeoylquinic acid, p‐coumaroylquinic acid, 5‐*O*‐caffeoylquinic acid, apigenin‐*O*‐pentoside, quercetin‐3‐*O*‐rutinoside, myricetin‐*O*‐rutinoside, quercetin‐3‐*O*‐glucoside, kaempferol 3′,4′‐di‐*O*‐rhamnoside, quercetin‐*O*‐pentoside, delphinidin‐3‐*O*‐glucoside, peonidin‐3‐*O*‐glucoside, malvidin‐3‐*O*‐glucoside, petunidin‐3‐(6′coumaroyl)‐glucoside. The presence of four anthocyanins in the extract was suggested, whereas in our study, no anthocyanin was detected.

**TABLE 1:** The chromatographic profile of the NaDES extract from RGP.

Compound	Concentration (mg/kg DW)
Gallic acid	3.12 ± 0.17
3,4‐dihydroxybenzoic acid	4.88 ± 0.22
2,5‐dihydroxybenzoic acid	5.85 ± 0.97
4‐dihydroxybenzoic acid	3.87 ± 0.11
Caffeic acid	4.14 ± 0.19
Syringic acid	< 1.35
*p*‐Coumaric acid	17.02 ± 1.09
Chlorogenic acid	31.42 ± 1.14
Sinapic acid	< 0.74
Ferulic acid	12.94 ± 1.07
Ellagic acid	< 1.00
Abscisic acid	< 0.83
Shikimic acid	< 0.85
Vanillic acid	236.36 ± 2.33
Abietic acid	25.01 ± 0.99
Azelaic acid	50.09 ± 1.02
Rosmarinic acid	2.35 ± 0.11
Sebacic acid	4.80 ± 0.44
Suberic acid	30.94 ± 1.07
Catechin	1.55 ± 0.10
Epicatechin	< 0.70
Epigallocatechin	2.60 ± 0.11
Taxifolin	< 0.79
Vitexin	19.62 ± 0.38
Isorhamnetin	1.23 ± 0.09
Naringin	< 0.73
Hesperidin	1.08 ± 0.09
Myricetin	8.88 ± 0.23
Rutin	1.23 ± 0.01
Quercetin	< 1.00
Kaempferol	< 0.98
Apigenin	< 0.82
Pinocembrine	< 0.85
Chrysin	0.89 ± 0.17
Galangin	< 0.85
t‐Resveratrol	20.66 ± 1.34
Isorhapontigenin	3.60 ± 0.87
Cis‐ Polidatin	32.05 ± 1.05
t‐Polidatin	< 1.04
Phlorizin	47.19 ± 1.14
Phloretin	0.49 ± 0.08
Procyanidin B2	< 0.85
Malvidin‐3‐O‐glucoside (Oenin)	< 0.64

### 3.2 Absorption, Distribution, Metabolism, and Excretion and Toxicity of the Identified Compounds in NaDES Extract

**FIGURE 1:**
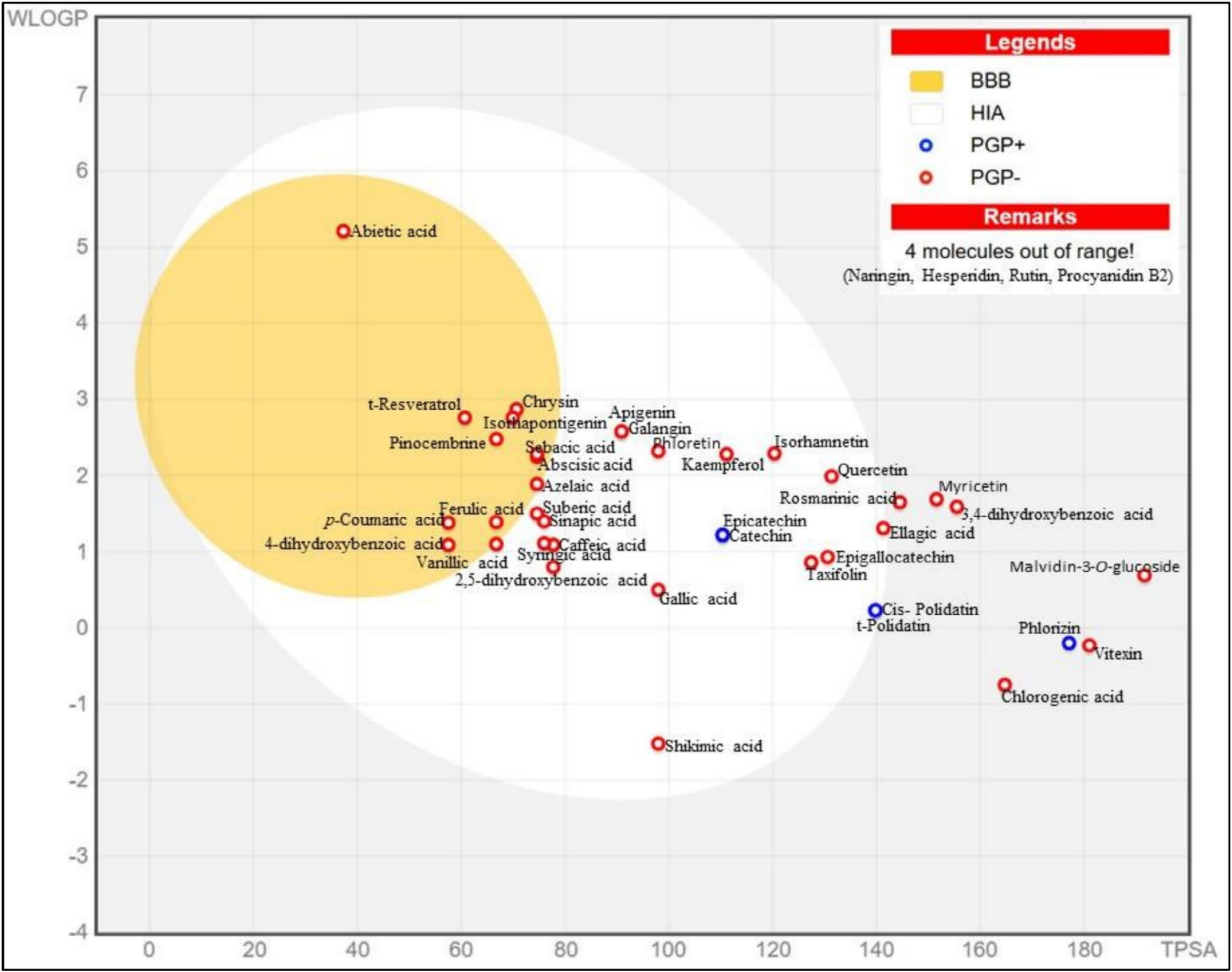
The BOILED‐Egg model prediction for the phytochemical compounds identified in the RDP NaDES extract. BBB, the blood–brain barrier; HIA, the human intestinal absorption; PGP+, P‐glycoprotein substrate; PGP−, non‐P‐glycoprotein substrate.

SwissADME and ProTox‐3.0 bioinformatic tools were employed to assess the absorption, distribution, metabolism, and excretion (ADME) as well as the oral and nutritional toxicity of the phytochemical compounds identified in the NaDES extract. This analysis aimed to predict and estimate the potential of the extract as an ingredient in functional food products. The theoretical and computational predicted models and parameters are presented in Figure 1, Table 2.

**TABLE 2:** Bioinformatic predictions for the phytochemical compounds identified in the RGP NaDES extract.

Signal molecules	Physicochemical properties (SwissADME)				BOILED‐egg model outputs (SwissADME)						Toxicity Prediction (ProTox‐3.0)				
											Oral			Nutritional	
	Molecular weight (g/mol)	Nr. Rotatable bonds	Nr. H‐bond acceptors	Nr. H‐bond donors	TPSA (Å^2^)	WLOGP (Log Po/w)	GI absorption	BBB permeant	P‐gp substrate	Bioavailability Score	LD_50_ (mg/kg)	Class	Accuracy	Pred.	Prob.
Gallic acid	170.12	1	5	4	97.99	0.50	High	−	−	0.56	2000	IV	70.97	−	0.83
3,4‐dihydroxybenzoic acid	308.24	2	8	6	155.52	1.59	Low	−	−	0.11	2000	IV	70.97	−	0.76
2,5‐dihydroxybenzoic acid	154.12	1	4	3	77.76	0.80	High	−	−	0.56	4500	V	100	−	0.84
4‐dihydroxybenzoic acid	138.12	1	3	2	57.53	1.09	High	+	−	0.85	2200	V	100	−	0.95
Caffeic acid	180.16	2	4	3	77.76	1.09	High	−	−	0.56	2980	V	70.97	−	0.77
Syringic acid	198.17	3	5	2	75.99	1.11	High	−	−	0.56	1700	IV	69.26	−	0.86
*p*‐Coumaric acid	164.16	2	3	2	57.53	1.38	High	+	−	0.85	2850	V	100	−	0.89
Chlorogenic acid	354.31	5	9	6	164.75	−0.75	Low	−	−	0.11	5000	V	69.26	−	0.64
Sinapic acid	224.21	4	5	2	75.99	1.40	High	−	−	0.56	1772	IV	70.97	−	0.80
Ferulic acid	194.18	3	4	2	66.76	1.39	High	+	−	0.85	1772	IV	70.97	−	0.82
Ellagic acid	302.19	0	8	4	141.34	1.31	High	−	−	0.55	2991	IV	70.97	+	0.71
Abscisic acid	264.32	3	4	2	74.60	2.25	High	−	−	0.55	5000	V	100	−	0.56
Shikimic acid	174.15	1	5	4	97.99	−1.52	High	−	−	0.56	9000	VI	69.26	−	0.64
Vanillic acid	168.15	2	4	2	66.76	1.10	High	−	−	0.85	2000	IV	69.26	−	0.87
Abietic acid	302.45	2	2	1	37.30	5.21	High	+	−	0.85	1000	IV	70.97	−	0.65
Azelaic acid	188.22	8	4	2	74.60	1.89	High	+	−	0.85	900	IV	100	−	0.80
Rosmarinic acid	360.31	7	8	5	144.52	1.65	Low	−	−	0.56	5000	V	68.07	−	0.65
Sebacic acid	202.25	9	4	2	74.60	2.28	High	+	−	0.85	900	IV	100	−	0.80
Suberic acid	174.19	7	4	2	74.60	1.50	High	−	−	0.85	900	IV	100	−	0.80
Catechin	290.27	1	6	5	110.38	1.22	High	−	+	0.55	10,000	VI	100	+	0.63
Epicatechin	290.27	1	6	5	110.38	1.22	High	−	+	0.55	10,000	VI	100	+	0.63
Epigallocatechin	306.27	1	7	6	130.61	0.93	High	−	−	0.55	10,000	VI	100	+	0.63
Taxifolin	304.25	1	7	5	127.45	0.86	High	−	−	0.55	2000	IV	100	+	0.63
Vitexin	432.38	3	10	7	181.05	−0.23	Low	−	−	0.55	832	IV	67.38	+	0.54
Isorhamnetin	316.26	2	7	4	120.36	2.29	High	−	−	0.55	5000	V	70.97	+	0.57
Naringin	580.53	6	14	8	225.06	−1.49	Low	−	+	0.17	2300	V	70.97	+	0.51
Hesperidin	610.56	7	15	8	234.29	−1.48	Low	−	+	0.17	12,000	VI	72.90	+	0.53
Myricetin	318.24	1	8	6	151.59	1.69	Low	−	−	0.55	159	III	100	+	0.63
Rutin	610.52	6	16	10	269.43	−1.69	Low	−	+	0.17	5000	V	100	+	0.54
Quercetin	302.24	1	7	5	131.36	1.99	High	−	−	0.55	159	III	100	+	0.63
Kaempferol	286.24	1	6	4	111.13	2.28	High	−	−	0.55	3919	V	70.97	+	0.66
Apigenin	270.24	1	5	3	90.90	2.58	High	−	−	0.55	2500	V	70.97	−	0.55
Pinocembrine	256.25	1	4	2	66.76	2.48	High	+	−	0.55	2000	IV	69.26	+	0.52
Chrysin	254.24	1	4	2	70.67	2.87	High	+	−	0.55	3919	V	70.97	−	0.55
Galangin	270.24	1	5	3	90.90	2.58	High	−	−	0.55	3919	V	70.97	+	0.66
t‐Resveratrol	228.24	2	3	3	60.69	2.76	High	+	−	0.55	1560	IV	68.07	−	0.89
Isorhapontigenin	258.27	3	4	3	69.92	2.76	High	+	−	0.55	2400	V	70.97	−	0.80
Cis‐Polidatin	390.38	5	8	6	139.84	0.23	High	−	+	0.55	1380	IV	68.07	−	0.60
t‐Polidatin	390.38	5	8	6	139.84	0.23	High	−	+	0.55	1380	IV	68.07	−	0.60
Phlorizin	436.41	7	10	7	177.14	−0.20	Low	−	+	0.55	3000	V	69.26	−	0.58
Phloretin	274.27	4	5	4	97.99	2.32	High	−	−	0.55	500	IV	69.26	−	0.74
Procyanidin B2	578.52	3	12	10	220.76	2.35	Low	−	−	0.17	2500	V	70.97	+	0.64
Malvidin‐3‐*O*‐glucoside	493.44	6	12	7	191.67	0.69	Low	−	−	0.17	5000	V	69.26	−	0.51

*Note:* “–” indicates negative; “+” indicates positive; LD_50_, median lethal dose, the dose at which 50% of test subjects die upon exposure to a compound, expressed in mg/kg body weight; toxicity classes and their corresponding colors are presented according to ProTox‐3.0, following the globally harmonized system for the classification and labeling of chemicals (GHS).

Abbreviations: BBB, blood–brain barrier; GI, gastrointestinal; P‐gp, P‐glycoprotein; TPSA, Topological Polar Surface Area; WLOGP, decadic logarithm of the *n*–octanol/water partition coefficient.

The physicochemical properties of the investigated compounds (Table 2), obtained from SwissADME, indicate a diverse structural profile within the extract. The molecular weights of these compounds range from 138.12 g/mol for 4‐dihydroxybenzoic acid to 610.56 g/mol for hesperidin, highlighting the presence of both low and high molecular weight phenolic compounds. The majority of the investigated compounds (90.70%) have a molecular weight below 500 g/mol, which, according to Lipinski's Rule of Five, suggests they are likely to exhibit good oral bioavailability in humans (Varma et al. 2006). Additionally, the topological polar surface area (TPSA) values varied significantly, ranging from 37.30 Å^2^ for abietic acid to 269.43 Å^2^ for rutin, reflecting considerable differences in molecular polarity. Furthermore, 72.09% of the compounds showed a TPSA of lower than 140 Å^2^ and < 10 rotatable bonds. According to Veber's Rules, these characteristics suggest that these compounds are likely to demonstrate good oral bioavailability, often providing a better predictive filter than Lipinski's Rule of Five (Soares et al. 2023).

The BOILED‐Egg (Brain Or IntestinaL EstimateD) permeation predictive model, shown in Figure 1, visually illustrates the human intestinal absorption (HIA), represented by the white region of the model, and the blood–brain barrier (BBB) penetration, represented by the yellow region (Daina and Zoete 2016). Only 23.23% of the investigated compounds were predicted to penetrate into the BBB. These compounds include 4‐dihydroxybenzoic acid, *p*‐coumaric acid, ferulic acid, abietic acid, azelaic acid, sebacic acid, pinocembrine, chrysin, trans‐resveratrol, and isorhapontigenin, suggesting that these compounds may exert neuroprotective or cognitive‐enhancing effects within the central nervous system. Conversely, limited BBB penetration provides an additional safety margin from a food application perspective by minimizing unintended central nervous system exposure (Obermeier et al. 2013). Additionally, 25.58% of the compounds, including 3,4‐dihydroxybenzoic acid, chlorogenic acid, rosmarinic acid, vitexin, naringin, hesperidin, myricetin, rutin, phlorizin, procyanidin B2, and malvidin‐3‐O‐glucoside, are positioned outside the HIA region and are predicted to demonstrate low gastrointestinal (GI) absorption with bioavailability scores between 0.11 and 0.56. These findings align with published literature reporting that flavonoid compounds generally require enzymatic deglycosylation or microbial metabolism before absorption in the small intestine or colon (Németh et al. 2003; Braune and Blaut 2016). Most of the compounds (81.40%) are predicted to be non‐P‐glycoprotein (P‐gp) substrates, with the exception of catechin, epicatechin, hesperidin, myricetin, rutin, phlorizin, and polidatin (cis and trans isomers), which are predicted to be potential P‐gp substrates. The P‐gp substrates compounds are subject to cellular efflux, making them less likely to exert local intestinal biological activity (Varma et al. 2006).

The ProTox‐3.0 toxicity predictions (Table 2) indicate that the investigated compounds have a safe profile. The lowest predicted median lethal dose (LD50), which is the amount at which 50% of test subjects die from exposure to a compound, was estimated at 159 mg/kg body weight for myricetin and quercetin, with 100% accuracy. This level falls within the III toxicity class, indicating that at concentrations between 50 < LD50 ≤ 300 mg/kg body weight, these compounds are considered toxic if swallowed. However, these predictions should be interpreted in the context of exposure levels, as the concentrations typically found in dietary intake are substantially lower than those associated with toxicological concern. Most of the other compounds are classified within toxicity classes IV to VI, indicating low acute toxicity to non‐toxicity and supporting a favorable safety profile. Furthermore, none of the investigated compounds fell within the highly toxic categories (I and II), suggesting no major acute toxicity concerns for food‐based applications under typical dietary exposure conditions.

Nutritional toxicity predictions showed predominantly negative outcomes for the phytochemical compounds, whereas several compounds (37.21%) were predicted to be potential nutritional toxic, with moderate probability ranging from 0.51 to 0.71. These *in silico* predictions highlight the importance of dose‐dependent evaluation and confirmation through experimental safety assessments.

From a food application perspective, the NaDES extract shows several advantageous characteristics related to the investigated compounds. The physicochemical profile of the identified phytochemical compounds indicates structural diversity that enables complementary biological effects, which are favorable for GI absorption. The polar compounds may exhibit reduced direct permeability but potential gut‐localized functionality. The coexistence of highly and poorly absorbed compounds could lead to dual functionality, such as rapid systemic antioxidant and anti‐inflammatory activity alongside sustained GI and microbiota‐modulating effects. Limited BBB permeability reduces potential systemic safety concerns, and the compounds predominantly fall within low acute toxicity classification predictions.

### 3.3 Inhibitory Mechanism of the Extract on α‐Amylase

The extract showed potent inhibition on α‐amylase, allowing us to estimate an IC_50_ value of 1.12 ± 0.12 mg GAE/mL (Table 3). The corresponding value for acarbose was 0.14 ± 0.01 mg/mL. Therefore, a higher extract concentration is needed for inhibition of 50% of enzyme activity. However, when expressed as inhibitory ratio (%) (Equation 1), a 99% inhibition was reached when adding 1.52 mg GAE/mL, suggesting a high inhibition rate.

**TABLE 3:** Stern–Volmer constant (*k*_SV_), the binding constant (*k*_*b*_), and the number of binding sites (*n*) of the heat treated enzymes quenched by NaDES extract from RGP.

Enzyme	Temperature (°C)	k_SV_ (×10^6^ Mol/L)	*k*_*q*_ (× 10^12^ mol L^−1^ s^−1^)	*k*_*b*_ (×10^6^ Mol/L)	*n*
α‐amylase	22	1.57 ± 0.55^A^	1.57 ± 0.55^A^	1.49 ± 0.42^A^	1.06 ± 0.05^A^
	37	1.31 ± 0.10^AB^	1.31 ± 0.10^AB^	1.27 ± 0.10^AB^	1.02 ± 0.08^A^
	45	1.12 ± 0.10^AB^	1.12 ± 0.10^AB^	1.10 ± 0.09^B^	1.04 ± 0.07^A^
	50	1.09 ± 0.16^AB^	1.09 ± 0.16^AB^	1.08 ± 0.09^B^	0.98 ± 0.01^AB^
	55	1.02 ± 0.08^B^	1.02 ± 0.08^B^	1.05 ± 0.04^B^	0.91 ± 0.02^B^
α‐glucosidase	22	1.32 ± 0.07^bc^	1.32 ± 0.07^bc^	1.18 ± 0.04^bc^	1.21 ± 0.07^A^
	37	1.09 ± 0.01^C^	1.09 ± 0.01^C^	1.05 ± 0.01^C^	1.26 ± 0.01^A^
	45	1.65 ± 0.16^A^	1.65 ± 0.16^A^	1.50 ± 0.14^A^	1.29 ± 0.06^A^
	50	1.48 ± 0.19^AB^	1.48 ± 0.19^AB^	1.36 ± 0.24^AB^	1.22 ± 0.01^A^
	55	1.21 ± 0.07^C^	1.21 ± 0.07^C^	1.08 ± 0.11^C^	1.17 ± 0.13^A^
Lipoxygenase	22	2.51 ± 0.01^D^	2.51 ± 0.01^D^	0.86 ± 0.17^C^	1.11 ± 0.04^C^
	37	2.29 ± 0.01^E^	2.29 ± 0.01^E^	0.71 ± 0.01^D^	1.25 ± 0.03^A^
	45	2.79 ± 0.02^B^	2.79 ± 0.02^B^	0.95 ± 0.02^B^	1.16 ± 0.10^B^
	50	2.93 ± 0.03^A^	2.93 ± 0.03^A^	1.04 ± 0.02^A^	1.10 ± 0.04^C^
	55	2.72 ± 0.11^C^	2.72 ± 0.11^C^	1.06 ± 0.07^A^	1.05 ± 0.02^D^

*Note:* Based on the Tukey test, different superscript uppercase letters indicate significant differences (*p* < 0.05) among results in the same column for each enzyme.

**FIGURE 2:**
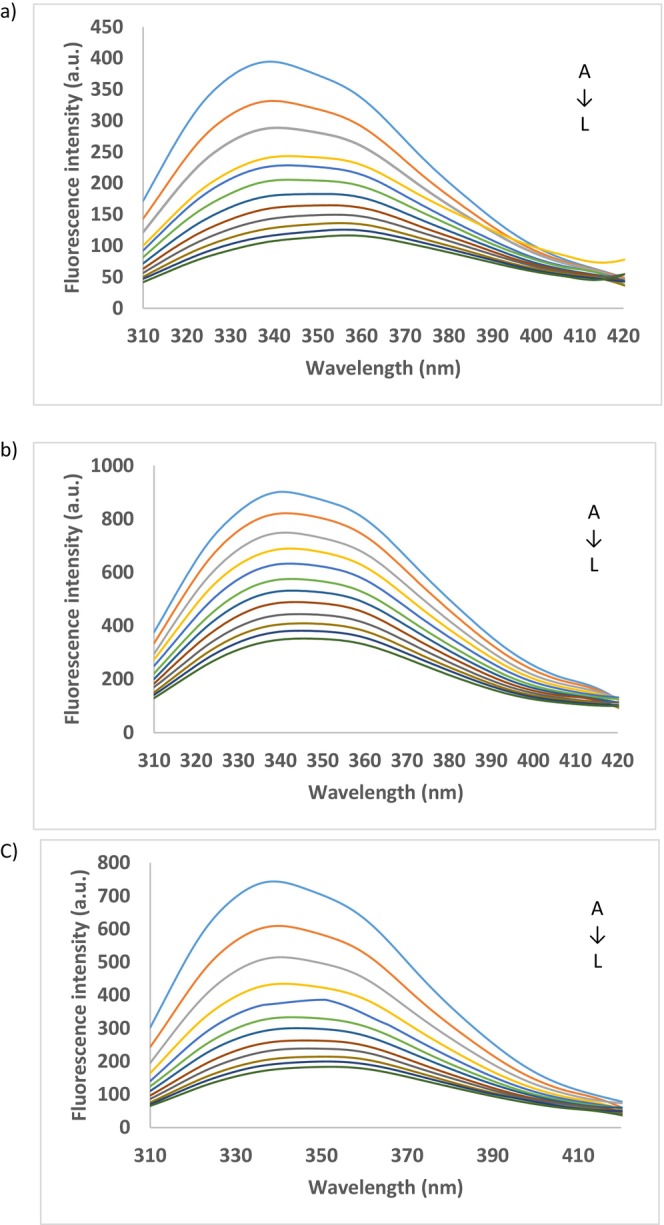
Fluorescence emission spectra of the interaction between α‐amylase (a), α‐glucosidase (b), and lipoxygenase (c) at 22°C with red grape pomace extract (from A (0) to L (1.23 × 10^−6^ Mol/L)).

In order to gain more structural details about small biomolecules binding to the α‐amylase, important information on the binding parameters and the change in the microenvironment of the fluorophore in the macromolecules can be provided by means of intrinsic fluorescence quenching. The approach is based on the sensitivity of tryptophan and tyrosine fluorescence properties to the polarity of the specific local environment (Vivian and Callis 2001). Therefore, upon successive titration of the enzymatic solution with extract in concentrations ranging from 0 to 1.2310^−6^ Mol/L GAE, a significant 18 nm red‐shift was found, suggesting the unfolding of the quaternary and tertiary structure of the enzyme, induced by the binding of small ligands. In general, red shifts in *λ*_max_ are associated with the extensive exposure of the previously buried hydrophobic residues, such as Trp and Tyr to the solvent molecules. Additionally, increasing the concentration of the extract up to 1.2310^−6^ Mol/L GAE caused the reducing of α‐amylase fluorescence, which was interpreted as the small bioactive from extract bound to the protein (Figure 2a).

In general, when heating, the *λ*_max_ red‐shifted with 11 nm, suggesting that the enzyme structure unfolds, thus becoming more prone for binding. The heat‐ and binding particularities of the enzyme molecule behavior allow us to divide the fluorescence quenching mechanism into static quenching and dynamic quenching, based on the binding constant values (Table 3). From Table 2 it can be observed that the *K*_*SV*_ values decreased with increasing temperature, from (1.57 ± 0.50) × 10^6^ L/Mol at 22°C to (1.02 ± 0.08) × 10^6^ L/Mol at 55°C. Regardless of the temperature, the Stern‐Volmer constants were significantly lower than the maximum value for the diffusion‐limited collisional quenching (*k*_*q*_) (2.0 × 10^10^ M^−1^ s^−1^) (Lakowicz 2006), thus indicating that the interactions between α‐amylase and RGP bioactive is a static, enthalpy‐driven process (Callis 2014) (Table 4).

**TABLE 4:** Thermodynamic parameters of the enzymes quenched by NaDES extract from RGP at different temperatures.

Enzyme	Temperature (°K)	ΔH (kJ/mol)	ΔS (J/mol/K)	ΔG (kJ/mol)
α‐amylase	295	1078.7	3.25	118.20
	310			69.37
	318			43.32
	323			27.04
	328			10.76
α‐glucosidase	295	3408.5	10.28	375.90
	310			221.70
	318			139.46
	323			88.06
	328			36.660
Lipoxygenase	295	−2373.8	−7.35	−202.92
	310			−92.54
	318			−33.66
	323			3.12
	328			39.91

In case of a static mechanism, the fluorescence data were analyzed to estimate the binding constant (*K*_b_) and the stoichiometry number (*n*) values for the complexes (Table 3). From Table 3, it can be observed that, *K*_*b*_ values decreased with increasing the temperature, whereas *n* values were around one, indicating the presence of at least one binding site able to bind near the hydrophobic sites of the enzyme. The corresponding *K*_b_ values were about 1.49 ± 0.42 × 10^6^ L/Mol at 22°C and 1.05 ± 0.11 × 10^6^ L/Mol at 55°C. Different values for binding constants are reported, considering the ligand‐protein structural relationship. For example, Aprodu et al. (2020) estimated significant higher values for the binding interaction between folic acid and bovine lactoferrin, suggesting values of 0.28 ± 0.01 × 10^8^ L/Mol for *K*_*SV*_ and 1.68 ± 0.01 × 10^8^ L/Mol for *K*_*b*_, respectively.

The intermolecular forces involved in the ligand‐protein complex formation may include hydrophobic forces, van der Waals interactions, electrostatic interactions, and hydrogen bonds (Ross and Subramanian 1981). To distinguish the contribution of each intermolecular energy to the complex formation, the standard Gibbs free energy change (*ΔG*), the standard enthalpy change (*ΔH*), and the standard entropy change (*ΔS*) of interactions were estimated, as described by Dumitrașcu et al. (2018). From Table 4 it can be seen that the binding mechanism of RGP bioactive to α‐amylase is complex, involving hydrophobic bonding, as indicated by the positive value of *ΔH* and *ΔS*. The kinetic and thermodynamic binding values indicated that the conformational selection model, as described by Du et al. (2016) can describe the enzyme‐ligand binding mechanism.

When analyzing more in depth, it seems that the native protein does not exist as a rigid conformation, but rather an ensemble of flexible conformational states (flexible fit), which coexist in equilibrium, so that ligands can selectively bind to the most suitable conformation, with the equilibrium shifting towards that binding state. However, as explained by Ross and Subramanian (1981), it is possible that the binding between α‐amylase and polyphenols from the extracts to follow a two‐step binding mode, involving van der Waals interactions and hydrogen bonding and partial immobilization, followed by hydrophobic interactions, leading to an inhibitory effect. Our results on the NaDES extract interaction with α‐amylase, a protein with a particular pharmacological interest, suggest that the polyphenols from RGP can act as antidiabetic molecules.

**FIGURE 3:**
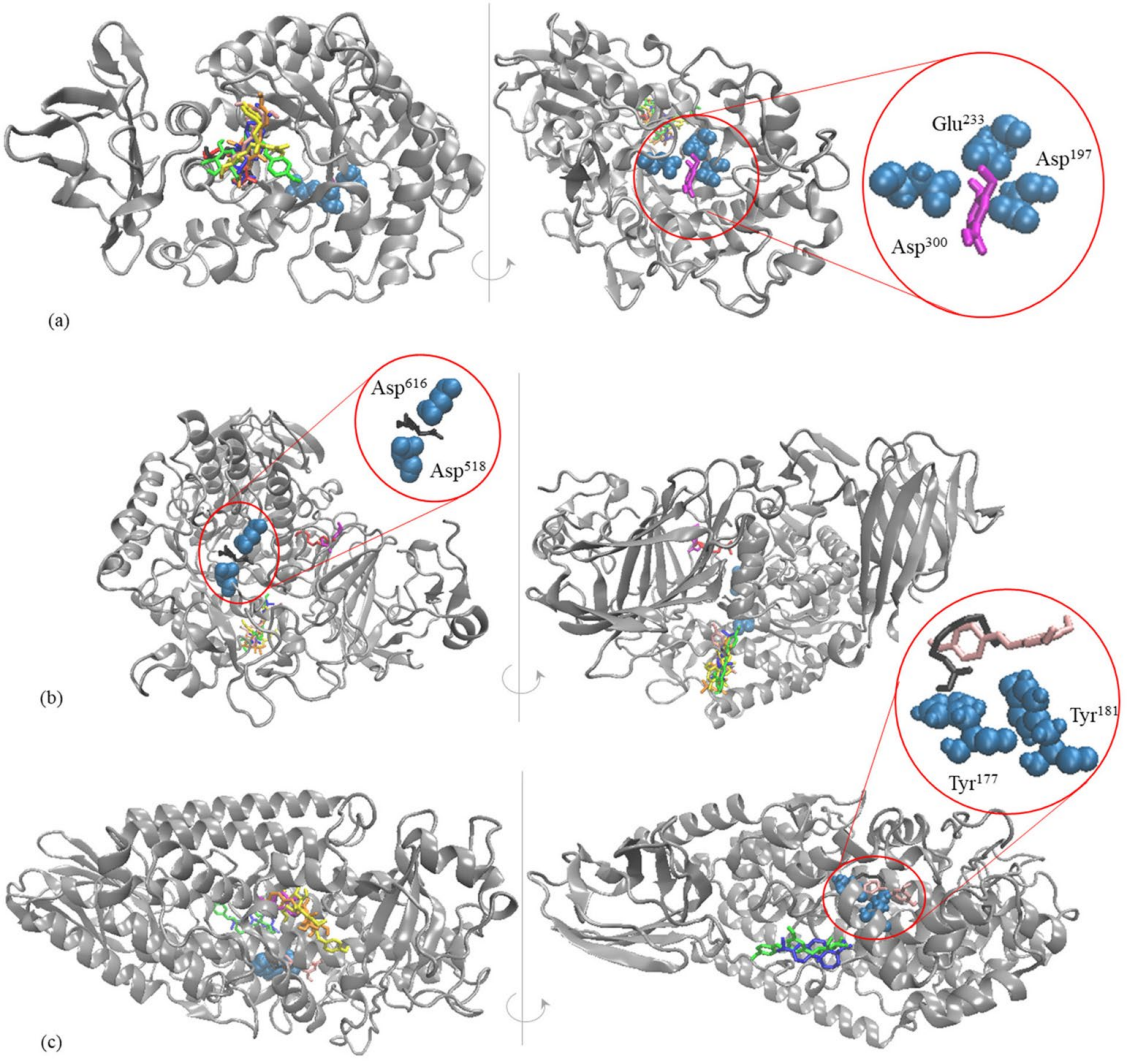
Superposition of the top scoring enzyme‐ligand models resulting from the *in silico* docking tests: complexes formed by α‐amylase (a), ɑ‐glucosidase (b), and lipoxygenase (c) with vanillic acid (magenta), azelaic acid (red), phlorizin (yellow), *cis*‐polydatin (green), chlorogenic acid (orange), suberic acid (black), abietic acid (blue), *t*‐resveratrol (pink). The enzymes, ligands, and catalytic amino acids are represented in NewCartoon, licorice, and Van der Waals style, respectively, by using the VMD software (Humphrey et al. 1996).

Additional details on the mechanism involved in the inhibition exerted by the phenolic compounds extracted from red grapes pomace on the α‐amylase activity were collected by analyzing the enzyme‐ligand complexes obtained from molecular docking tests. The enzyme appeared to accommodate most of the tested ligands, with rather low affinity, in a shallow cavity located on the surface of the molecule, the free interaction energy values ranging from −0.2 to 1.7 kcal/mol (Table 5, Figure 3). The only exception concerned vanillic acid, which exerted high affinity (ΔG_int_ of −0.8 kcal/mol) towards the substrate binding site. In particular, vanillic acid directly interfaced the Asp^197^, Glu^233^, and Asp^300^ residues from the active site, which are pivotal for the catalytic activity of human pancreatic α‐amylase (Axer et al. 2021). The complex formed by α‐amylase with vanillic acid appeared thermodynamically stable (ΔG_diss_ of 2.8 kcal/mol), suggesting that no spontaneous detachment of the molecules might occur. The hydrogen bonds with Arg^195^ and Glu^233^, ionic interaction with His^305^, hydrophobic contacts with Trp^58^, and π‐staking interaction with Tyr^62^ are mainly involved in the stabilization of the interaction surface. Our results regarding the potential interference of vanillic acid with the catalytic activity of α‐amylase comply with the observations of previous studies investigating the inhibitory mechanism of various phytochemicals. In particular, malvidin‐3‐glucoside (Serea et al. 2022) and gallocatechin gallate (Wu et al. 2018) were previously reported to overlap the active site of the enzyme, with downstream effect on hydrolytic activity.

**TABLE 5:** Atomic level details on the contacts between the molecules of complexes formed by α‐amylase, α‐glucosidase, and lipoxygenase with vanillic acid, azelaic acid, phlorizin, *cis*‐polydatin, chlorogenic acid, suberic acid, abietic acid, t‐resveratrol.

Ligand		α‐amylase	α‐glucosidase	Lipoxygenase
Vanillic acid	ΔG_int_, kcal/mol	−0.8	−1.0	0.8
	ΔG_diss_, kcal/mol	2.8	3.2	1.2
	Interfacing residues	Trp^58^, Tyr^62^, Leu^162^, Arg^195^, Asp^197^, Ala^198^, His^201^, Glu^233^, Ile^235^, His^299^, Asp^300^, Gly^306^	His^263^, Leu^264^, Ser^265^, Pro^266^, Leu^269^, Ser^270^, Thr^274^, Ile^276^, Gly^288^	Val^243^, Thr^366^, Agr^370^, Ser^447^, Leu^448^, Phe^450^, Ala^453^, Tyr^470^, Gln^549^
	Amino acids interacting with ligands	H bonds: Arg^195^, Glu^233^ Ionic interaction: His^305^ HyC: Trp^58^ π‐staking interaction: Tyr^62^	H bonds: Ser^270^, Gly^288^ Hydrophobic contacts: His^263^, Leu^269^, Ile^276^	H bonds: Arg^370^, Thr^545^ Hydrophobic contacts: Leu^448^, Phe^450^, Gln^549^
Azelaic acid	ΔG_int_, kcal/mol	0.2	0.5	−0.5
	ΔG_diss_, kcal/mol	0.4	0.2	1.2
	Interfacing residues	Thr^11^, Arg^252^, Ser^289^, Asp^290^, Pro^332^, Gly^334^, Phe^335^, Arg^398^, Asp^402^	Gly^259^, Leu^260^, Ala^261^, His^263^, Ser^265^, Pro^266^, Leu^267^, Leu^269^, Ile^276^, Pro^629^, Glu^630^, Gln^633^	Tyr^181^, Gln^363^, Thr^364^, His^367^, Leu^368^, Leu^414^, Ile^415^, Leu^420^, Phe^421^, Ala^424^, Asn^425^, Trp^599^, His^600^, Ala^603^, Leu^607^
	Amino acids interacting with ligands	H bonds: Ser^289^, Pro^332^, Asp^402^, Gly^403^ Ionic interactions: Arg^252^, (2) Arg^398^, Arg^421^ Hydrophobic contacts: Thr^11^, Phe^335^, Arg^398^	H bonds: Leu^264^ Hydrophobic contacts: His^263^, Leu^269^, Ile^276^, Gln^633^	H bonds: Gln^363^, Asn^425^ Ionic interactions: His^367^, His^372^ Hydrophobic contacts: Leu^368^, Ile^415^, Phe^421^, Ala^603^ Leu^607^
Phlorizin	ΔG_int_, kcal/mol	0.2	−0.3	0.7
	ΔG_diss_, kcal/mol	1.2	1.6	1.0
	Interfacing residues	Tyr^2^, Ser^3^, Pro^4^, Thr^11^, Arg^252^, Phe^335^, Asp^402^	Pro^194^, Leu^195^, Glu^196^, Ile^581^, Arg^585^	Val^243^, Arg^246^, Arg^370^, Ser^447^, Ala^453^, Arg^457^
	Amino acids interacting with ligands	H bonds: Tyr^2^, Arg^10^, Thr^11^, Arg^252^, Pro^332^, Thr^336^, Gly^334^, Arg^398^ Hydrophobic contacts: Phe^335^	H bonds: Leu^195^, Tyr^609^ Hydrophobic contacts: Pro^194^, Leu^496^, Thr^578^, Ile^581^	H bonds: Arg^246^, Asp^285^, Arg^370^, Arg^457^, Tyr^470^, Glu^549^ Hydrophobic contacts: Leu^344^, Val^361^, Ala^453^
cis‐polydatin	ΔG_int_, kcal/mol	−0.2	−0.4	0.5
	ΔG_diss_, kcal/mol	1.8	1.8	0.8
	Interaction surface, Å^2^	78.6	31.9	50.9
	Interfacing residues	Pro^4^, Arg^10^, Thr^11^, Arg^252^, Gly^334^, Phe^335^, Arg^398^, Asp^402^	Pro^194^, Leu^195^, Glu^196^, Leu^565^	Gly^174^, Asn^180^, Lys^183^, Gln^611^
	Amino acids interacting with ligands	H bonds: Arg^10^, Ser^289^, Arg^291^, Pro^332^, Asp^402^, Arg^421^ Cation‐π interactions: Arg^252^ Hydrophobic contacts: Tyr^2^, Pro^4^, Thr^11^, Asp^290^ π‐stacking interaction: Phe^335^	H bonds: Gly^605^, Phe^490^ Hydrophobic contacts: Leu^195^, Glu^196^, Ile^581^, Tyr^609^	H bonds: Asn^180^, Lys^183^, Ala^606^, Gln^609^, Gln^611^ Hydrophobic contacts: Gln^611^, Asn^613^
Chlorogenic acid	ΔG_int_, kcal/mol	0.6	−0.7	2.4
	ΔG_diss_, kcal/mol	0.8	0.0	−1.0
	Interfacing residues	Tyr^2^, Ser^3^, Pro^4^, Arg^252^, Asp^290^, Phe^335^	Pro^194^, Phe^490^, Thr^491^, Leu^565^, Leu^571^, Thr^578^, Ile^581^	Val^243^, Arg^370^, Ser^447^, Ala^453^, Ile^454^, Arg^457^, Tyr^470^, Gln^549^
	Amino acids interacting with ligands	H bonds: Thr^6^, Gln^7^, Ser^289^, Pro^332^, Gly^334^, Arg^398^ Hydrophobic contacts: Pro^4^, Phe^335^	H bonds: Glu^196^, Thr^491^ Hydrophobic contacts: Pro^194^, Leu^565^, Leu^577^, Thr^578^, Ile^581^	H bonds: Thr^545^ Ionic interactions: Arg^246^ Hydrophobic contacts: Phe^450^, Ala^453^, Phe^544^
Suberic acid	ΔG_int_, kcal/mol	1.7	−1.1	−0.8/−1.0
	ΔG_diss_, kcal/mol	−0.8	2.2	2.8
	Interfacing residues	Thr^11^, Pro^332^, Gly^334^, Phe^335^, Arg^398^, Asp^402^, Gly^403^, Arg^421^	Trp^376^, Asp^404^, Ile^441^, Trp^481^, Trp^516^, Asp^518^, Met^519^, Arg^600^, Trp^613^, Asp^616^, Asp^645^, Phe^649^, His^674^	Phe^177^, His^367^, Leu^368^, His^372^, Ala^410^, Leu^414^, Ile^415^, Leu^607^, Ile^673^, Fe
	Amino acids interacting with ligands	H bonds: Gly^403^ Ionic interactions: Arg^252^, Arg^398^, Arg^421^ Hydrophobic contacts: Thr^11^, Phe^335^	H bonds: His^674^ Hydrophobic contacts: Trp^376^, Phe^649^	Ionic interactions: (2) His^367^, His^372^ Hydrophobic contacts: Gln^363^, Leu^368^, Leu^414^, Ile^415^, Phe^421^, Ala^410^
Abietic acid	ΔG_int_, kcal/mol	0.0	−1.0	−0.5
	ΔG_diss_, kcal/mol	0.2	1.2	0.7
	Interfacing residues	Pro^4^, Thr^6^, Gln^7^, Gln^8^, Arg^10^, Thr^11^, Arg^252^, Ser^289^, Asp^290^, Pro^332^, Gly^334^, Phe^335^, Asp^402^	Pro^194^, Leu^195^, Glu^196^, Thr^491^, Leu^565^, Leu^577^, Ile^581^, Gly^605^, Tyr^609^	Gly^174^, Asn^180^, Lys^183^, Trp^605^, Ala^606^, Gln^609^, Gln^611^, Glu^612^
	Amino acids interacting with ligands	H bonds: Pro^332^ Ionic interactions: Arg^252^, Arg^398^ Hydrophobic contacts: Pro^4^, Thr^11^, Phe^335^	H bonds: Thr^491^ Hydrophobic contacts: Pro^194^, Leu^195^, Leu^577^, Thr^578^, Ile^581^, Tyr^609^	H bonds: Ala^606^ Hydrophobic contacts: Gln^611^, Lys^183^, Gln^609^, Trp^605^
Resveratrol	ΔG_int_, kcal/mol	0.3	−1.0	−0.6/−1.2
	ΔG_diss_, kcal/mol	0.9	2.2	3.0
	Interfacing residues	Pro^4^, Thr^11^, Arg^252^, Ser^289^, Asp^290^, Pro^332^, Gly^334^, Phe^335^, Arg^398^, Asp^402^	Ile^193^, Pro^194^, Leu^195^, Phe^311^, Leu^313^, Val^358^, Leu^574^, Leu^577^, Thr^578^, Ile^581^, Phe^603^, Ala^604^, Gly^605^	Tyr^181^, Gln^363^, His^367^, Leu^368^, His^372^, Ala^410^, Leu^414^, Ile^415^, Phe^421^, Asn^425^, Ala^603^, Leu^607^, Fe
	Amino acids interacting with ligands	H bonds: Pro^332^, Ser^298^ Hydrophobic contacts: Pro^4^, Thr^11^	H bonds: Val^193^, Pro^194^, Leu^496^, Leu^565^, Leu^574^, Leu^577^, Thr^578^, Ile^581^, Phe^603^	H bonds: His^600^ Hydrophobic contacts: Tyr^181^, Leu^368^, Leu^414^, Phe^421^, Ala^603^, Leu^607^

*Note:* ΔG_int_ and ΔG_diss_ are the free energy associated to receptor‐ligand interaction and dissociation, respectively.

### 3.4 Inhibitory Mechanism of the Extract on α‐Glucosidase

When comparing with α‐amylase, the NaDES extract showed a significantly higher inhibitory potential on α‐glucosidase, with an IC_50_ value of 0.47 ± 0.03 mg GAE/mL (Table 3), whereas the corresponding value for acarbose was 0.72 ± 0.01 mg/mL. Therefore, a lower extract concentration is needed for the inhibition of 50% of enzyme activity when compared with acarbose. The extract was able to inhibit 99% of α‐glucosidase activity at a concentration of 1.90 mg GAE/mL. The results suggest that the extract shows potent antidiabetic activity.

The fluorescence emission spectra of α‐glucosidase at 22°C treated with increased concentration of extract are shown in Figure 2b. Similarly, with α‐amylase, given the gradual addition of RGP extracts, the fluorescence intensity of α‐glucosidase gradually decreased. Therefore, increasing the TPC concentration from 0 to 1.23 × 10^−6^ Mol/L GAE, the relative fluorescence intensity gradually decreased from 100% to ~61% (Figure 2b). Heating caused unfolding of the enzyme conformation, with a maximum red‐shift of 5 nm, from 340 nm at 22°C to 346 nm at 55°C. When quenching, the maximum extent of 6.5 nm red‐shift in *λ*_max_ was found, suggesting the unfolding of polypeptide chains, allowing the exposure of previously buried hydrophobic amino acids.

The Stern‐Volmer and binding constants are given in Table 3, together with the number of binding sites. At 22°C, the binding constant value were of 1.18 ± 0.04 × 10^6^ Mol/L, whereas increasing the temperature lead to an initial decrease, to 1.05 ± 0.01 × 10^6^ Mol/L at 37°C, followed by an increase up to 1.50 ± 0.14 × 10^6^ Mol/L at 45°C and further decreases. The increase–decrease of the *K*_*b*_ values may suggest a temperature mediated flexible fit between the enzyme and polyphenols from the extracts. Anghel et al. (2023) observed the same trend in the binding constant values as depending on temperature, suggesting significantly lower value of 25.09 ± 2.14 × 10^−2^ Mol/L for the GP extract obtained by S/L UAE using 70% ethanol. These differences may be due to the different extraction conditions, allowing the increase in the anthocyanin's content in the extract. The value of binding sites suggested that polyphenols from the extracts had one inhibition site or a single class of inhibition site on α‐glucosidase. Based on the positive values of *ΔH* and *ΔS*, it may be assume that the major interactions involved in protein‐ligand binding are hydrophobic. When comparing the kinetic and thermodynamic parameters, it seems that the small biomolecules from RGP showed a higher affinity for α‐glucosidase when compared with α‐amylase, whereas the binding process was enthalpy driven (Table 4).

The ability of the major phenolic compounds extracted from the red grapes to recognize and bind to different sites on the α‐glucosidase molecule was also tested through the *in silico* investigations. Analyzing the results presented in Table 5, Figure 3 one can observe that the ligands preferentially attached to three different cavities located on the protein surface. Out of the tested ligands, of particular interest is suberic acid, which interacts with the catalytic GH31 domain of the α‐glucosidase molecule, being in direct contact with Asp^518^ and Asp^616^ residues, which are responsible for the catalytic nucleophile and acid/base activity of the enzyme (Roig‐Zamboni et al. 2017; Serea et al. 2022). This *in silico* observation supports the experimental results regarding the inhibitory potential of the phenolic compounds from red grape pomace on α‐glucosidase activity, suggesting the antidiabetic activity of suberic acid. In addition to the good affinity between molecules, as indicated by the ΔG_int_ of −1.1 kcal/mol, the α‐glucosidase—suberic acid complex appeared stable from the thermodynamic point of view (ΔG_diss_ of 2.2 kcal/mol).

Roig‐Zamboni et al. (2017) provided atomic level details on the high‐resolution models of α‐glucosidase in complex with different active site directed iminosugar inhibitors, such as 1‐deoxynojirimycin and its N‐hydroxyethyl‐ derivative, which are pharmacological chaperones. They explained that the complexes were stabilized through hydrogen bonds involving the side‐chains of Asp^404^, Asp^518^, Arg^600^, Asp^616^, and His^674^ and hydrophobic contacts with Trp^376^, Ile^441^, Trp^516^, Met^519^, Trp^613^, and Phe^649^ (Roig‐Zamboni et al. 2017). Our results are in good agreement with the experimental observations of Roig‐Zamboni et al. (2017), as all amino acids involved in the interaction with 1‐deoxynojirimycin were found in the present *in silico* experiment to be in direct contact with suberic acid (Table 5).

Our results are as well supported by Panigrahy et al. (2020), who reported that suberic acid is a major compound found in the *Hedychium coronarium* Koen. extract, which exerts high inhibitory activity towards α‐amylase and α‐glucosidase. Moreover, Ofosu et al. (2022) showed that fermentation with *Pediococcus acidilactici* of the pressurized cooked sorghum increased the level of suberic acid, while significantly enhancing the α‐glucosidase inhibition.

As presented in Figure 3b, except for suberic acid, all other ligands are either accommodated by a pocket of the N‐terminal β‐sheet domain (vanillic acid and azelaic acid) or by a cavity at the confluence of N‐terminal β‐sheet and GH31 domains (phlorizin, cis‐polydatin, chlorogenic acid, abietic acid, and resveratrol). Although the attachment of these ligands to the enzyme surface does not impede substrate binding, as no direct participation of the catalytic amino acids was observed, some local structural rearrangement might occur; therefore, potentially interfering with the hydrolytic activity.

### 3.5 Inhibitory Mechanism of the Extract on Lipoxygenase

The effect of RGP extract on lipoxygenase fluorescence intensity is shown in Figure 2c, which indicates the shift in enzyme emission maximum when adding different concentrations of extract. Regardless of the temperature, lipoxygenase showed a *λ*_max_ of 339 nm, with no significant differences in fluorescence intensity. On titration with RGP extract, the fluorescence intensity decreased regularly with increasing the concentration of the extract, due to a variety of molecular interactions, such as molecular rearrangements, excited‐state reactions, ground state complex formation, energy transfer, and collisional quenching, indicating interactions between bioactive compounds from the extract and lipoxygenase.

When quenching, significant red‐shifts were observed in a temperature dependent manner. The increase in temperature from 22°C to 37°C led to less flexible molecules, with red‐shifts of 6 and 4 nm, respectively, suggesting local rearrangements in protein structure which led to a lower affinity for ligands. Increasing the temperature up to 55°C followed by quenching led to significant red‐shifts of 12 nm at 45°C, 7.5 nm at 50°C, and 10.5 nm at 55°C, indicating a more heat‐induced flexible molecule, prone to bind small molecules from the extract.

The intrinsic fluorescence data were correlated with the inhibitory capacity of the extract. Therefore, the IC_50_ value was 0.06 ± 0.01 mg GAE/mL, whereas for quercetin the corresponding value was significantly higher at 5.17 ± 0.07 μg/mL. However, the extract showed a high inhibition rate, a concentration of 0.4 mg GAE/mL leading to the inhibition of about 82% of the initial enzyme activity.

The binding parameters for RGP to lipoxygenase are given in Table 3. Two mechanisms of binding between small molecules and proteins are possible, according to the *K*_*SV*_ constant dependence on temperature: a static and dynamic quenching mechanism, respectively. For the static quenching, quenching rate constants decreased with increasing temperature, but the contrary effect was seen in the case of dynamic quenching (Du et al. 2016). Based on data shown in Table 3, the mechanism of binding is static up to 37°C and dynamic at higher temperatures. From the value of *n* (Table 3), it was found that there is at least one independent class of binding sites on lipoxygenase for the extract bioactive, which indicates that more than one extract molecule is bound to one enzyme molecule.

The *K*_*b*_ values followed a temperature induced decrease–increase trend, which means that the interactions between polyphenols from the extract and lipoxygenase are temperature mediated, suggesting a flexible fitting between molecules. The thermodynamic parameters indicated the involvement of van der Waals forces and/or hydrogen bonds in the temperature range of 37°C–50°C, due to the negative value of *ΔH* and *ΔS* (Table 4).

The molecular docking results involving the lipoxygenase as a receptor for the major phenolic compounds from the red grape pomace indicated that the tested ligands attach with different affinities to three different sites of the enzyme (Figure 3c, Table 5). The complexes formed by lipoxygenase with suberic acid and resveratrol are of particular importance, as the ligands are able to recognize and interact with amino acids of the active site and the catalytic iron. The particular attachment of any of the two ligands obstructs the access channel to the catalytic iron, as suberic acid is in direct contact with Phe^177^, while resveratrol with Tyr^181^, whose side chains are positioned inward the cavity (Gilbert et al. 2011). Both ligands are in close vicinity and each interact with three of the four amino acids engaged in iron coordination, namely His^367^, His^372^, His^550^, and Ile^673^ (Table 5). Moreover, suberic acid and resveratrol established hydrophobic contacts with the Leu^414^ residues, which controls the O_2_ access in the vicinity of the substrate (Gilbert et al. 2011). These observations imply the direct involvement of the suberic acid and resveratrol in hampering the substrate transformation by lipoxygenase.

Although the other ligands do not bind in the vicinity of the active site, their contribution to enzyme inhibition should not be neglected, even at low concentration. Because of the intrinsic instability of lipoxygenase (Gilbert et al. 2011), even small spatial reorganization of the cavities accommodating the ligands might result in alteration of the catalytic activity. Indeed, Mahesha et al. (2007) reported the non‐competitive inhibitory activity of soy isoflavones, which attach to different sites of lipoxygenase compared to the substrate. Mahesha et al. (2007) also suggested a second lipoxygenase inhibition mechanism, consisting of the ability of the phenolic acids to scavenge the free radicals, a process which provides electrons to the Fe^3+^, turning the enzyme to the resting state.

## 4 Conclusions

From the data reported in this study, the bioactive from red grape pomace appear to be promising food‐derived extracts that potently inhibited α‐amylase, α‐glucosidase, and lipoxygenase. The chromatographic profile revealed the predominance of phenolic acids in the extract, probably highly dependent on the solvent and extraction conditions applied. The *in silico* predictions support the potential application of red grape pomace NaDES extract as functional food ingredients, including incorporation into beverages, nutraceutical formulations, antioxidant‐enriched products, and clean‐label preservative systems. It is important to note that while theoretical and computational predictions provide valuable preliminary insights into safety profiles, these models do not account for potential synergistic or antagonistic interactions among extract constituents, matrix effects within complex food systems, or chronic dietary exposure. The natural deep eutectic solvent extract allowed obtaining a high inhibition rate of the enzymes, with half‐minimal inhibitory concentration lower than the corresponding values for the common drug, except for α‐glucosidase. The intrinsic fluorescence investigations evidenced a static binding mechanism, suggesting a temperature mediated flexible fit of the enzyme molecules to ligands. The main forces involved in interactions were hydrogen, van der Waals, and hydrophobic interactions. The molecular docking tests suggested that vanillic acid is mainly responsible for the α‐amylase inhibitory properties, whereas suberic acid inhibits the α‐glucosidase activity; therefore, it explains the anti‐diabetic activity exerted by the extract from red grape pomace. In the case of lipoxygenase, the direct inhibitory mechanism might be related to suberic acid and resveratrol interaction with the catalytic iron and key amino acids form the active site of the enzyme. Further validation through in vivo studies is required to confirm the enzyme inhibition, bioaccessibility, and long‐term safety.

## Author Contributions

**Nicoleta Balan:** data curation, methodology, validation, visualization, writing – original draft, formal analysis. **Ilir Mërtiri:** methodology, investigation, formal analysis, visualization, writing – original draft, software, data curation. **Iuliana Aprodu:** investigation, methodology, validation, visualization, writing – original draft, writing – review and editing, formal analysis. **Silviu Măntăilă:** investigation, writing – original draft, methodology, visualization, formal analysis, validation. **Gabriela Râpeanu:** funding acquisition, validation, visualization, writing – original draft, resources. **Nicoleta Stănciuc:** conceptualization, investigation, funding acquisition, methodology, visualization, validation, resources, project administration, supervision, writing – review and editing, writing – original draft. **Elisabeta Irina Geana:** formal analysis, visualization, validation, methodology, writing – original draft.

## Funding

This work was supported by a grant from the Ministry of Research, Innovation and Digitization, CNCS‐UEFISCDI, project number PN‐IV‐P1‐PCE‐2023‐0129, within PNCDI IV, 9PCE/08.01.2025.

## Conflicts of Interest

The authors declare no conflicts of interest.

## Data Availability

Data are contained within the article.
